# Oxygen Exposure and Tolerance Shapes the Cell Wall-Associated Lipids of the Skin Commensal *Cutibacterium acnes*

**DOI:** 10.3390/microorganisms11092260

**Published:** 2023-09-08

**Authors:** Iuliana Popa, David Touboul, Tilde Andersson, Eduardo Fuentes-Lemus, Cyrille Santerre, Michael J. Davies, Rolf Lood

**Affiliations:** 1 Analytic and Biological Lipid Systems (Lip(Sys)2), Pharmacy Department, University Paris-Saclay, Bâtiment Henri Moissan, 91400 Orsay, France; 2 CNRS, Institut de Chimie des Substances Naturelles, UPR 2301, University Paris-Saclay, Avenue de la Terrasse, 91198 Gif-sur-Yvette, France; david.touboul@cnrs.fr; 3 CNRS, Laboratoire de Chimie Moléculaire (LCM), Institut Polytechnique de Paris, University Paris-Saclay, Route de Saclay, 91120 Palaiseau, France; 4 Department of Clinical Sciences Lund, Division of Infection Medicine, Lund University, SE-221 00 Lund, Sweden; tilde.andersson@uzh.ch; 5 Department of Biomedical Sciences, University of Copenhagen, Blegdamsvej 3, 2200 Copenhagen, Denmark; eduardo.lemus@sund.ku.dk (E.F.-L.); davies@sund.ku.dk (M.J.D.); 6 Institut Supérieur International de la Parfumerie, de la Cosmétique et de l’Arôme Alimentaire (ISIPCA), 34-36 rue du Parc de Clagny, 78000 Versailles, France; csanterre@isipca-lafabrique.fr

**Keywords:** *Cutibacterium acnes*, ceramides, fatty acids, lipids, mono-, di- diacylglyceryl, oxidation, oxidative stress, protein carbonylation, skin, sulfoquinozyldiacylglycerol

## Abstract

*Cutibacterium acnes* is one of the most abundant bacteria on the skin. Being exposed to oxygen and oxic stress, the secretion of the bacterial antioxidant protein RoxP ensures an endogenous antioxidant system for the preservation of skin health. To investigate the impact of the antioxidant RoxP on oxidation of the bacteria, wildtype and an isogenic *roxp* mutant were cultured in anaerobic and oxic conditions. The carbonylated status of proteins were recorded, as were the most significant modifications in a relative intensity of free fatty acids (FFA) and lipids containing fatty acids (FA), such as di- (DG) and triglycerides (TG), di- (DGDG) and sulfoquinozyldiacylglycerol (SQDG) and ceramides. Concerning the fatty acid types, it was observed that the free fatty acids contained mainly C12:0–C26:0 in hydroxy and acylated forms, the DG contained mainly C29:0–C37:0, the TG contained mainly C19:0–C33:0, and the DGDG/SQDGs contained very long fatty acids (C29:0–C37:0) demonstrating the interdependence of de novo synthesis of lipids and RoxP. The area of DGDG peaks (924.52, 929.56 and 930.58) were affected by bacterial growth conditions, with the exception of *m*/*z* 910.61. Moreover, the FFA unsaturation is wider in the SQDG species (C30:0 to C36:6) than in DG, TG or free FFA species. It could be concluded that both environmental oxidative statuses, as well as the prevalence of bacterial antioxidant systems, significantly shape the lipidome of *C. acnes*.

## 1. Introduction

*Cutibacterium acnes* is a predominant skin commensal, also highlighted as an opportunistic pathogen in several pathologies (e.g., acne, low-grade chronic inflammation on prostheses) [1,2,3]. The commensal nature of the bacterium has been attributed to certain clades of *C. acnes,* while others are highly associated with specific diseases (e.g., acne) [4]. Being a skin commensal, the facultative anaerobe *C. acnes* is constantly exposed to oxygen and oxic stress. One of the mechanisms to cope with oxygen is through the secretion of the antioxidant protein RoxP, facilitating colonization of the skin [5]. It was shown that in the case of skin dysbiosis such as actinic keratosis or basal cell carcinoma, the *C. acnes* phylotype IA is less prevalent, and in particular in actinic keratosis, the quantity of RoxP is lower on the skin [6].

This protein, besides protecting the neighboring host human stratum corneum from oxidative exposure, facilitates growth and colonization of *C. acnes* [6] together with putative lipases, which releases specific surface lipids [3].

Changes in *C. acnes* surface lipids, and modification of sebum lipids released from sebaceous gland and environmental changes [3,7] trigger structural and immunological changes in skin and in pilosebaceous follicles [8]. Despite the critical nature of RoxP for survivability of *C. acnes* on the skin, and its favorable effects for its host’s health, including prevention or defense against oxidative stress, UV irradiation, and skin aging, little information is available on the physiological effect of RoxP on the bacterium itself. To our knowledge, there is limited information on the influence of *C. acnes* in general on skin constitutive lipids and proteins, including their oxidation or degradation during aging, pollution or disease (i.e., atopies) [3,7].

The aim of the study was to examine the hypothesis that physiological oxygen levels could modulate the composition of lipids in *C. acnes*, and that presence of the bacterial antioxidant RoxP in itself can affect lipid composition and carbonylation levels regardless of physiological oxygen levels.

## 2. Materials and Methods

### 2.1. Bacterial Strains and Cultivation

*C. acnes* isolate KPA171202 and its isogenic *roxp* mutant (KPA171202Δ*roxp*) were grown at 37 °C under anaerobic conditions in Wilkins–Chalgren anaerobe broth (WC) until reaching exponential phase (2 days). The cultures were diluted (1:1000) and spread over Tryptic Soy Broth Agar plates. The plates were placed either in anaerobic or oxic conditions, at 37 °C, and incubated until full lawns were visible (3–7 days). All colonies were harvested, washed in PBS three times, and stored at −20 °C until further processing.

### 2.2. Determination of Protein Concentration

Working solutions were prepared daily by mixing 50 parts of reagent A (sodium carbonate, sodium bicarbonate, bicinchoninic acid and sodium tartrate in 0.1 M sodium hydroxide) with 1 part of reagent B (CuSO_4_, 4%), as indicated by the manufacturer (Thermo Fisher Scientific, Waltham, MA, USA). The BCA (bicinchoninic acid) working solutions (200 μL) were pipetted into the wells of a 96-well plate, and 25 μL of samples were added, giving a BCA working solution: sample ratio of 8:1. The wells were quickly placed in the reader and shaken for 15 s in a smooth motion at 37 °C before incubation for 30 min at 37 °C. The absorbance was measured at 562 nm using a ClarioStar microplate reader (BMG Labtech, Ortenberg, Germany)

### 2.3. Quantification of Protein Carbonyls

Quantification of protein carbonyls was carried out as described by Hawkins et al. [9]. Briefly, 50 µL of sample was aliquoted into Eppendorf tubes (1.5 mL) adding 50 µL 10 mM DNPH (dinitrophenylhydrazine) in 2.5 M HCl, or 50 µL of 2.5 M HCl (control). The solutions were vortexed and incubated for 15 min in the dark at 21 °C before addition of 25 µL of 50% *w*/*v* TCA. This solution was kept at −20 °C for 20 min and was then centrifuged at 9000× *g* for 15 min at 4 °C. The supernatant was removed, and the protein pellets were washed twice with ice-cold ethanol/ethyl acetate (1:1) and centrifuged (2 min, 9000× *g*) between washes. After the final wash, samples were dried at 30 °C under vacuum before re-dissolving the pellet in 100 µL of 6 M guanidine HCl. The absorbance of the samples was recorded at 370 nm using a SpectraMax i3 plate reader (Molecular Devices, San Jose, CA, USA). Concentrations were determined using the extinction coefficient of DNPH at 370 nm (22,000 M^−1^ cm^−1^), with the carbonyl concentration (in nmoles mL^−1^) calculated by multiplying the value = [(Abs at 370 nm)/22,000] by 1 × 10^6^. The values were then normalized against the protein concentration (in mg mL^−1^) to express the carbonyl concentration as nmol of carbonyl per mg protein.

### 2.4. Lipids Purification and Fractionation

*C. acnes* (wildtype and isogenoic *roxp* mutant) pellets (300 mg) were suspended into glass tubes in 7 mL PBS (pH 7.4). Then, the cell pellets were homogenized by ultrasonic treatment, then 20 mL of chloroform–methanol (1/1) were added. The whole lipids extract was obtained after two sequential partitioning steps [10]. Half of the obtained lipids of each sample were fractionated by a LC-NH2 method into neutral lipids, ceramides, neutral glycolipids, free fatty acids and neutral phospholipids and anionic compounds [11]. The obtained lipid fractions were spotted on HPTLC silica gel 60 plates (Camag, Muttenz, Switzerland), migrated versus the specific standards in specific solvents and revelated by specific chemical reagents [12]. In the HPTLC of fatty acids and the neutral lipids, the spotted amount of the samples is 50% of the whole amount of the specific lipid fraction versus the spotted standard (triglycerides-TG, diglycerides-DG, monoglycerides-MG, fatty acids-FA, acylated and hydroxy fatty acids, (Sigma, Burlington, MA, USA) of 5 µL (2 mg/mL). The migration of both specific lipid fractions in the HPTLC was made in hexane/diethylether/acetic acid (70/30/1, (vol/vol)). The visualization was made for both HPTLC in Cu acetate (3%) in 8 M solution of H_3_PO_4_ at 150 °C [12]. The fatty acids distribution from the HPTLC plate was assessed by scanning the plate with a Chromatoscan CS-930 (Shimadzu, Tokyo, Japan) similar to previous HPTLC work assay [12] as a preliminary lipid identification. Half of the total lipids of each sample was used for MS lipid analysis.

### 2.5. MS Lipids Analysis

All lipid standards were dissolved in methanol and diluted to the required concentrations for spiking. Lipids including TG (11:1–11:1–11:1), DG (8:0–8:0), MG (15:1), PC (10:0–10:0), LPC (13:0), PE (10:0–10:0), LPE (14:0), PS (10:0–10:0), LPS (17:1), PG (10:0–10:0), SM (d18:1–12:0), provided by Sigma Chemicals, Cer (d18:1–12:0) and dCer (d18:1–12:0) Cer1P (d18:1–12:0), provided from Matreya (Nanterre, France), were used as standards.

### 2.6. SFC-HRMS Analysis

Qualitative profiles of lipids were obtained using a 1260 Infinity Analytical SFC system (Agilent Technologies, Waldbronn, Germany) coupled with a 6540 Q-ToF mass spectrometer (Agilent Technologies, Waldbronn, Germany) fitted with electrospray (ESI dual JetStream, Agilent Technologies, Waldbronn, Germany). The SFC system is composed of a controller module (G1170A), a SFC module (G4301A), a binary pump (G4302A), a degasser (G4225A), an auto sampler (G4303A), which was thermostated at 4 °C and equipped with a 5 µL injection loop, a thermostatically controlled column compartment (G1316C), a UV-DAD detector (G1315C), a back pressure regulator BPR (G4301A), and a make-up isocratic pump (G1310B). T-union was placed in front of the BPR in order to split the mobile phase into two fluxes, one to BPR and the other to MS. A caloratherm preheater (Sandra Selerity Technologies, Kortrijk, Belgium) set at 60 °C was installed at the entrance of the ion source to avoid the freezing of the mobile phase.

The experimental conditions of SFC-HRMS have been previously optimized and published [13,14]. Briefly, SFC parameters were fixed for BPR at 130 bar and 60 °C. The mobile phase consisted of CO_2_ (solvent A) and co-solvent (solvent B, 20 mM ammonium acetate in methanol—ethanol (1:1, *v*/*v*)). The program was as follows: 0 min, 1% B; 1.5 min, 4% B; 2.5 to 5.5 min, 15% B; 7.5 min, 30% B; and 8.5 to 15 min, 45% B. A make-up solvent was added between the analytical column and the BPR. The make-up pump delivered a mixture of 20 mM ammonium acetate in methanol—ethanol (1:1, *v*/*v*) at 200 μL/min from 0 to 6 min, then 100 μL/min from 7 to 19 min. This make-up solvent is added to the column in order to transport the molecules to the ion source and to improve the ionization efficiencies of all eluted compounds. The samples were injected in full loop 5 µL with overfill factor 3 and separated on diethanolamine-packed column (Torus DEA, 2.1 mm × 100 mm × 1.7 µm, Waters, Wexford, Ireland). Each lipid fraction was dissolved in chloroform—methanol (1:3, *v*/*v*) at a concentration of 0.1 mg/mL. The temperature of the column oven was fixed at 60 °C with a flow rate of 0.9 mL/min.

Mass spectrometry (MS) parameters were set as follows: Drying gas temperature was 350 °C, Vcap 3500 V. Full-scan mode was employed in a range of *m*/*z* 50–1700 at 2 GHz, giving a mass resolution higher than 25,000 at *m*/*z* 922. Calibration solutions containing two internal reference compounds, purine C_5_H_4_N_4_ at *m*/*z* 121.0509 and HP-921 (hexakis (1H, 1H, 3H-tetrafluoropentoxy) phosphazene) C_18_H_18_O_6_N_3_P_3_F_24_ at *m*/*z* 922.0098, were continuously introduced resulting in mass accuracy below 5 ppm.

### 2.7. Data Processing and Statistical Analysis

SFC-MS data were obtained and processed using Agilent Mass Hunter Workstation Data Acquisition software MRM data B.08.00 on the target lipids, including the *m*/*z* of precursor and product ions, and the retention time were exported using Qualitative Analysis B.08.00 software (Agilent Technologies, Santa Clara, CA, USA). Next, an in-house database constructed using the Skyline software package (MacCoss Laboratory, University of Washington, Seattle, WA, USA) was applied to determine the peak area of assigned lipids from replicate raw data. Sample lipid amounts in the MS spectra were normalized by the whole lipid pool per injection.

## 3. Results

### 3.1. The Proteome Carbonylation Is Affected by Presence of RoxP

**Figure 1:**
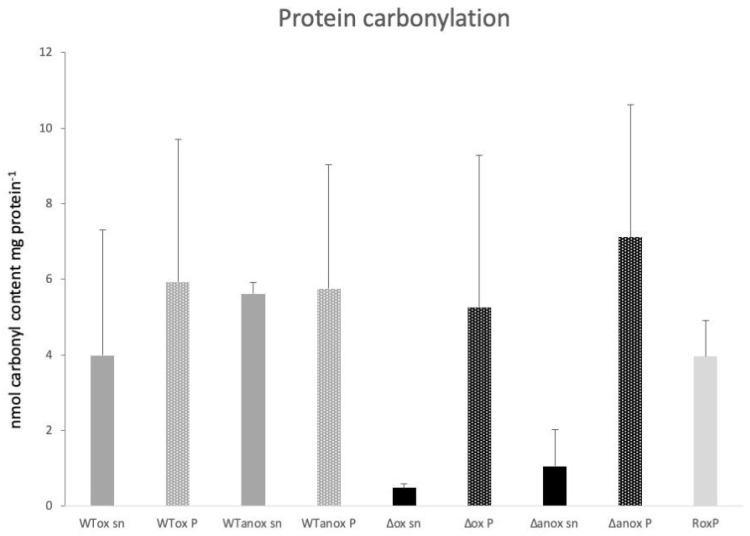
Degree of carbonylation motifs in the bacterial proteomes. Material from wildtype (WT) and the isogenic *roxp* mutant (Δ) *C. acnes* were cultivated in oxic (ox) and anoxic (anox) conditions, after which the secretome (sn) and the cellular (P) fractions were analyzed for carbonylation. The data were collected from two biological replicates, with two technical duplicates in each set, with the error bars showing the standard deviation. All material was normalized to 1 mg of protein.

In order to investigate the impact of oxygen for general carbonylation of the proteome, *C. acnes* was cultured in oxic and anoxic conditions, and carbonyl groups were labeled. No significant differences could be detected in the carbonylated protein content in the wildtype bacteria, regardless of growth condition (+/− oxygen) or fraction (extracellular vs. cellular material), Figure 1. In the isogenic *roxp* mutant, a trend in carbonylation reduction could be detected in the extracellular fraction, both under oxic and anoxic conditions (*p* = 0.11). Purified RoxP has in itself a high degree of carbonylation (Figure 1).

### 3.2. Free Fatty Acid Changes in Bacteria Lacking the Antioxidant RoxP

**Figure 2:**
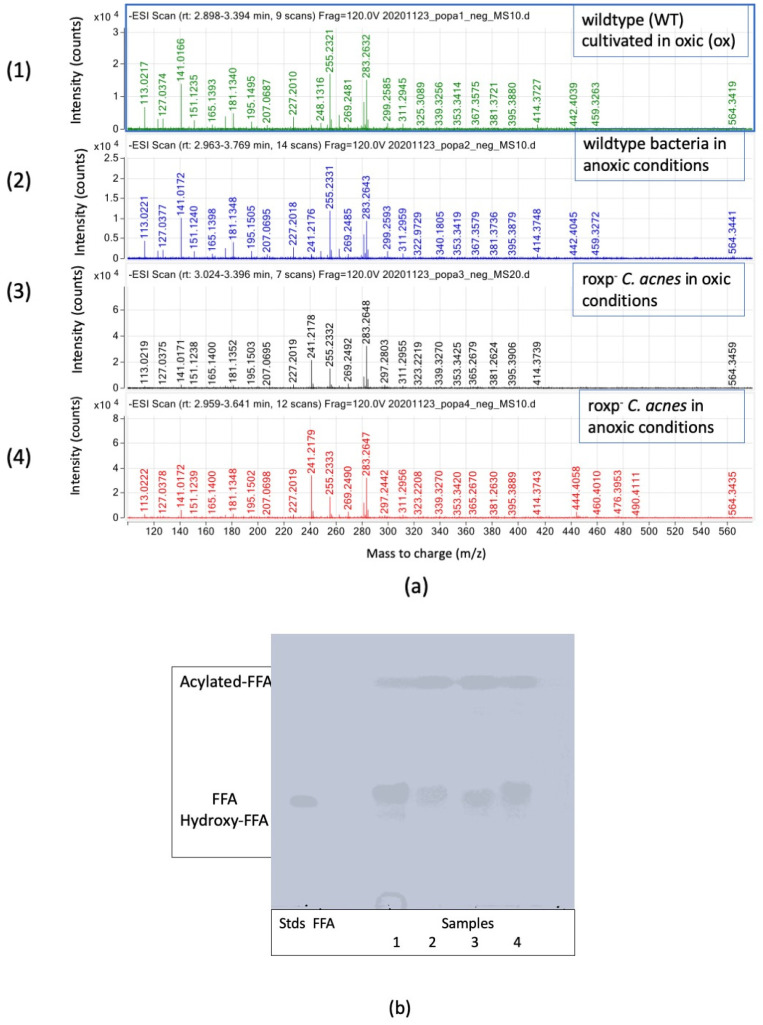
Fatty acid species detection in *C. acnes*. (**a**) Fatty acids species identified by SFC-HRMS in negative mode; intensity (count) versus mass-to charge (*m*/*z*). Analyzed samples are wildtype bacteria cultured in oxic (1) or anoxic (2) conditions, compared to the *roxp*^−^ isogenic mutant cultured in oxic (3) or anoxic (4) conditions. (**b**) HPTLC of the fatty acid fractions for the same samples. Spotted amount of the samples are 50% of the whole amount. Spotted standard of fatty acid is 5 µL (2 mg/mL). Visualization in Cu acetate [12].

Free fatty acids were analyzed with MS running (Figure 2a) in the negative mode, as well as detected through HPTLC (Figure 2b), demonstrating the presence of C12:0–C26:0 fatty acids, as well as certain species that were partly unsaturated (Supplementary Table S1). Free fatty acids were more abundant (76.40%) in wildtype bacteria compared to the *roxp* isogenic mutant lacking the antioxidant RoxP (36.2%) (Figure 2b and Table 1), while the trend for acetylated fatty acids (Acyl FFAs) or Hydroxy fatty acids (OH-FFAs) were not as clear. Specifically, there is a lack of ions with *m*/*z* 141.01 in *roxp*^−^ samples, while there was a higher abundancy of ions with *m*/*z* 241.21. Meanwhile, ions with *m*/*z* 255.23 are more prevalent in RoxP-containing strains, regardless of culture conditions (e.g., oxic vs. anoxic).

### 3.3. Complex Lipids Profile of C. acnes Cultivated in Oxic and Anoxic Conditions Vary Based on Presence of RoxP

The lipid detection from wildtype (WT) and the isogenic *roxp* mutant (Δ) *C. acnes* cultivated in oxic (ox) and anoxic (anox) conditions by SFC-HRMS in negative mode is given in Figure S1 as a function of the retention time. Lipid classes such as triglycerides (TG) (retention time—0.6–1.2 min), diglycerides (DG) 2 ± 0.3 min), ceramides (Cer) (retention time—3.5 min) and MGDG (retention time—5.4 ± 0.02 min) were identified as shown for the *C. acnes* lipidome [15]. Besides minor differences in peak intensity of several lipid classes, minor polar lipids such as mono-, di- and sulfoquinozyldiacylglycerol (MGDG, DGDG, SQDG) were observed too. Also, an increase in the relative intensities (counts) of triglycerides in *roxp^−^* bacteria cultured in oxic conditions was observed versus the specific mass to charge (Figure S1).

#### 3.3.1. Modified Diglyceride Relative Intensities (Counts) Are Positively Affected by Presence of the Antioxidant RoxP

**Figure 3:**
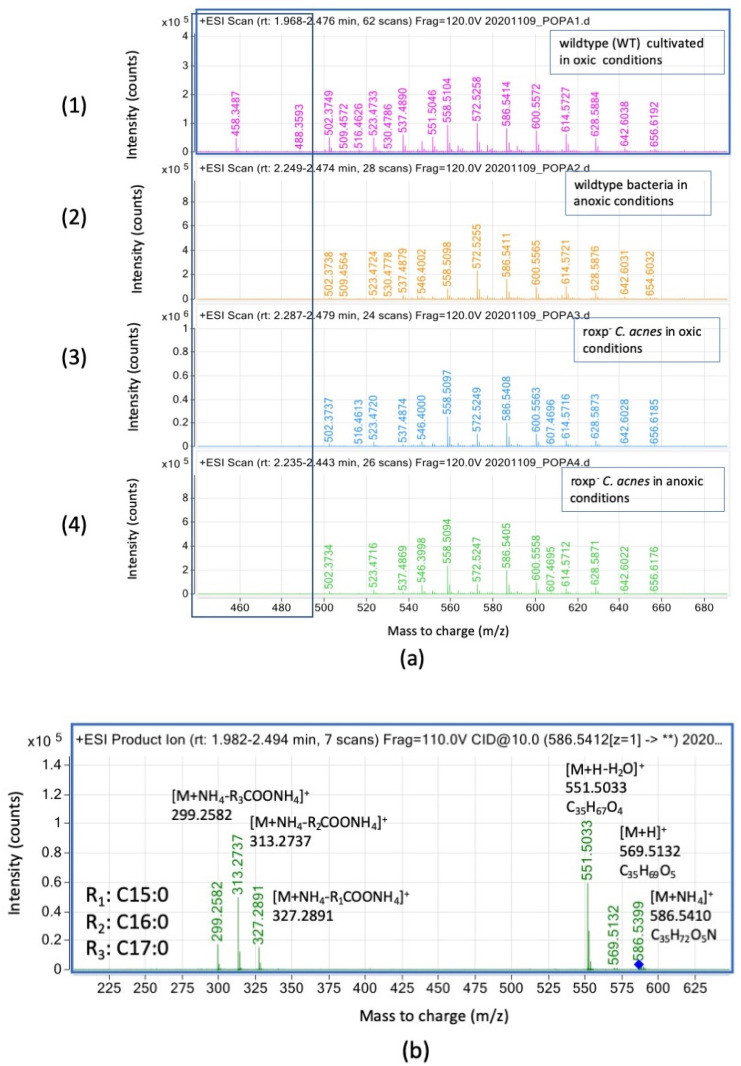
Diglyceride (DG) species detection in *C. acnes* by SFC-HRMS in positive mode. Intensity (count) versus mass-to charge (*m*/*z*); (**a**) samples analyzed are wildtype bacteria cultured in oxic (1) or anoxic (2) conditions, compared to the *roxp*^−^ isogenic mutant cultured in oxic (3) or anoxic (4) conditions. (**b**) MS2 fragmentation of the precursor ion at *m*/*z* 586.54 (DG C32:0) (see the blue diamond) with a collision energy of 10 eV.

Neutral lipids (monoglycerides, diglycerides, and triglycerides) were separated using HPTLC, demonstrating a reduction in abundance of DGs in *roxp*^−^ strains, with a following increase in MGs, disregarding cultivation conditions (Figure S2). Specifically, modified DGs were only present in wildtype bacteria. DGs contained very long fatty acids, ranging from C29:0 to C37:0 (Table S2), and varied in composition between the different samples (Figure 3a). Specifically, ions with *m*/*z* 458.34 could only be detected in wildtype bacteria grown under oxic conditions. Further, *m*/*z* 572.52 were more abundant in wildtype bacteria as compared to the *roxp^−^* mutant (Figure 3a).

For confirmation of the structure, the DG C32:0 with *m*/*z* 586.54 was fragmented in MS2 and three characteristic fragments were obtained (*m*/*z* 327.28–C17:0; *m*/*z* 313.27–C16:0; and *m*/*z* 299.25–C15:0 (Figure 3b)). Therefore, DG C32:0 can be annotated as a mixture of DG C16:0/C16:0 together with DG C15:0/C17:0.

#### 3.3.2. Triglyceride Relative Intensities (Counts) Are Negatively Affected in the Presence of Oxygen upon Lack of Functional Bacterial Antioxidant System

**Figure 4:**
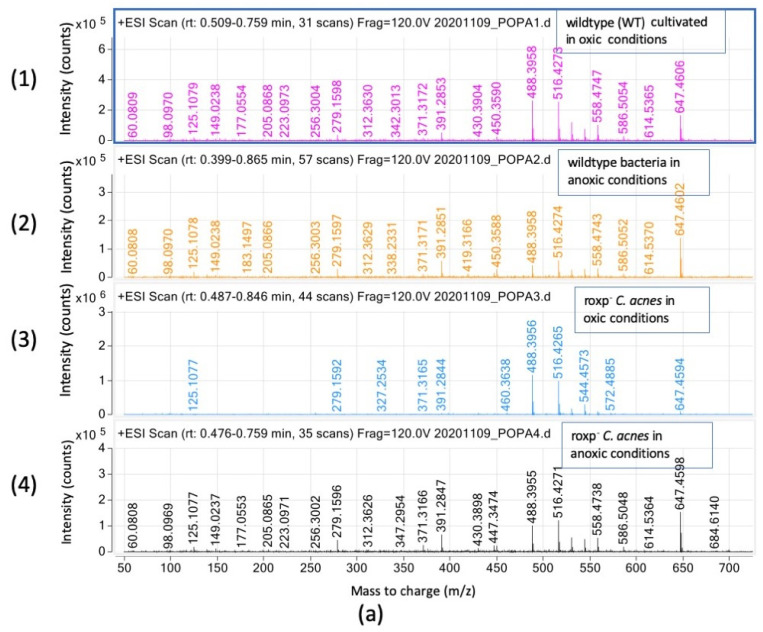

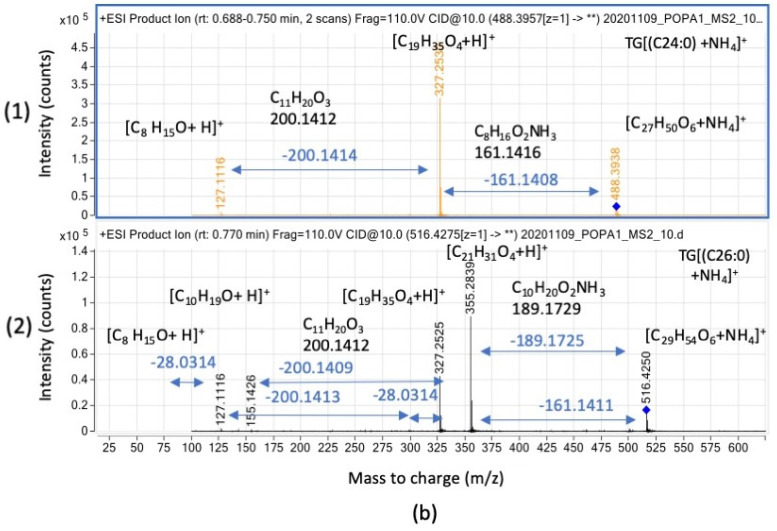
Triglyceride (TG) species detection in *C. acnes* by SFC-HRMS in positive mode. Intensity (count) versus mass-to charge (*m*/*z*); (**a**) samples analyzed are wildtype bacteria cultured in oxic (1) or anoxic (2) conditions, compared to the *roxp*^−^ isogenic mutant cultured in oxic (3) or anoxic (4) conditions. (**b**) MS2 fragmentation of the precursor ion at *m*/*z* 488.3957 (TG C24:0) (1) and *m*/*z* 516.4275 (TG C26:0) (see the blue diamond) (2) with a collision energy of 10 eV (detail in blue color for the size of the fragments).

To further investigate the prevalence of triglycerides and their specific composition in the samples, we analyzed the TG to determine its dependency upon oxic conditions as well as bacterial antioxidant systems (e.g., RoxP). The intensity of certain species with specific *m*/*z* ions displayed a significant oxic/anoxic dependency, with *m*/*z* 488.39 and *m*/*z* 516.42 being more prevalent in bacteria cultured under oxic conditions (Figure 4a). Conversely, *m*/*z* 391.28 and *m*/*z* 647.46 were more abundant in bacteria cultured under anoxic conditions. Strikingly, bacteria lacking the antioxidant RoxP and still exposed to oxygen, lacked a significant number of TGs (Figure 4a). The TG possessed long fatty acids (C19:0–C33:0; Table S3). After fragmentation of two major peaks (C24:0, *m*/*z* 488.39 and C26:0, *m*/*z* 516.42), several minor fragments related to fatty acids C8:0 and C10:0 were detected (Figure 4b, Table S3), suggesting that shorter fatty acids are present in the TG composition, as already described in the literature [16].

#### 3.3.3. Presence of Antioxidants Is Critical for High Relative Intensities of DGDG and SQDG in *C. acnes*

**Figure 5:**
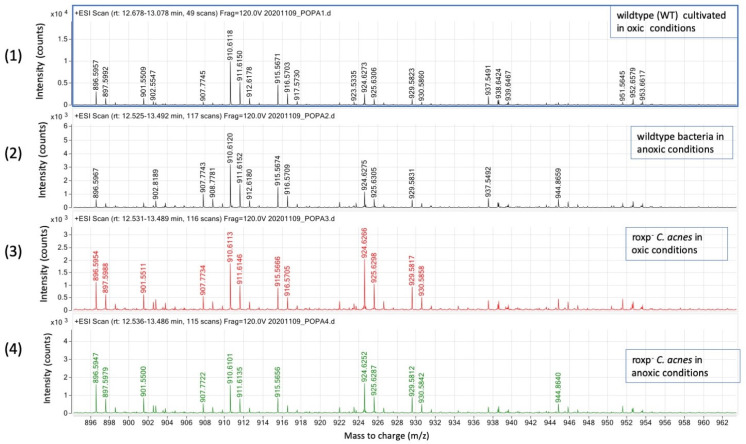
DGDG species detection in *C. acnes* by SFC-HRMS in positive mode. Intensity (count) versus mass-to charge (*m*/*z*); samples analyzed are wildtype bacteria cultured in oxic (1) or anoxic (2) conditions, compared to the *roxp*^−^ isogenic mutant cultured in oxic (3) or anoxic (4) conditions.

**Figure 6:**
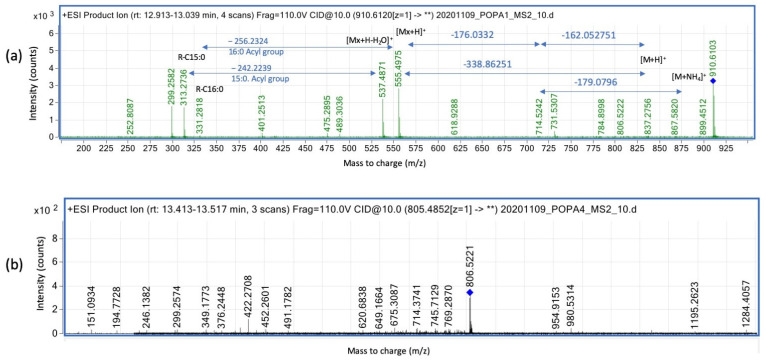
MS2 fragmentation of DGDG and SQDG ions. (**a**) MS2 fragmentation of *m*/*z* 910.612 (see the blue diamond) (DGDG) with an energy of 10eV (detail in blue color for the size of the fragments), and (**b**) MS2 fragmentation of the precursor ion at *m*/*z* 806.5221 (SQDG) (see the blue diamond) with a collision energy of 10 eV.

SQDGs and DGs were found by SFC-HRMS analyzing the material eluted between 13.3 and 13.6 min (Figure 5). In lipids sample of *roxp^−^* bacteria in oxic and anoxic condition, ion peaks for *m*/*z* of 924.52, 929.56 and 930.58 are enhanced (Figure 6 and Table 2). Further, wildtype bacteria have a trend to have less relative abundancy of higher *m*/*z* ions compared to the *roxp*^−^ strains, with peaks *m*/*z* 910.61, 915.56, 924.62 and 929.58 varying according to ca 1: 0.5: 0.25: 0.15 in wildtype bacteria, and ca 1: 0.5: 1: 0.5 in the RoxP mutant (extracted from Table 2). Specifically, a significant modification of the DGDGs peak area could be seen in the *roxp*^−^ mutant, regardless of the cultivation status, as exemplified using *m*/*z* 910.61, 915.56, 924.62 and 929.58 (Table 2). The *m*/*z* identified for SQDG (Table S4) were compared with those from the literature (Table S5) [15], resulting in species annotation of C30:0 (*m*/*z* 789.48), C31:0 (*m*/*z* 791,40), C32:6 (*m*/*z* 805.42), C33:6 (*m*/*z* 819:43), C34:6 (*m*/*z* 833.45), C34:6 (*m*/*z* 826.57), C35:6 (*m*/*z* 842.51) and C36:6 (*m*/*z* 856.53).

MS2MS2 fragmentation was performed for ion *m*/*z* 910.61 on the DGDG (Figure 6a) and for the ion *m*/*z* 806.52 in SQDG (Figure 6b) species. It was found that the DGDG ion of 910.61 contained C15:0/C16:0 in an acyl conformation, and the SQDG ion of 806.52 contained acyl fatty acid length C17:1/16:0. The fragmentation of both ions showed good parity with that obtained before by Lopes et al. (Table S5) [17].

#### 3.3.4. Ceramide Species Are Mainly Unaffected by Oxic Stress in *C. acnes*

**Figure 7:**
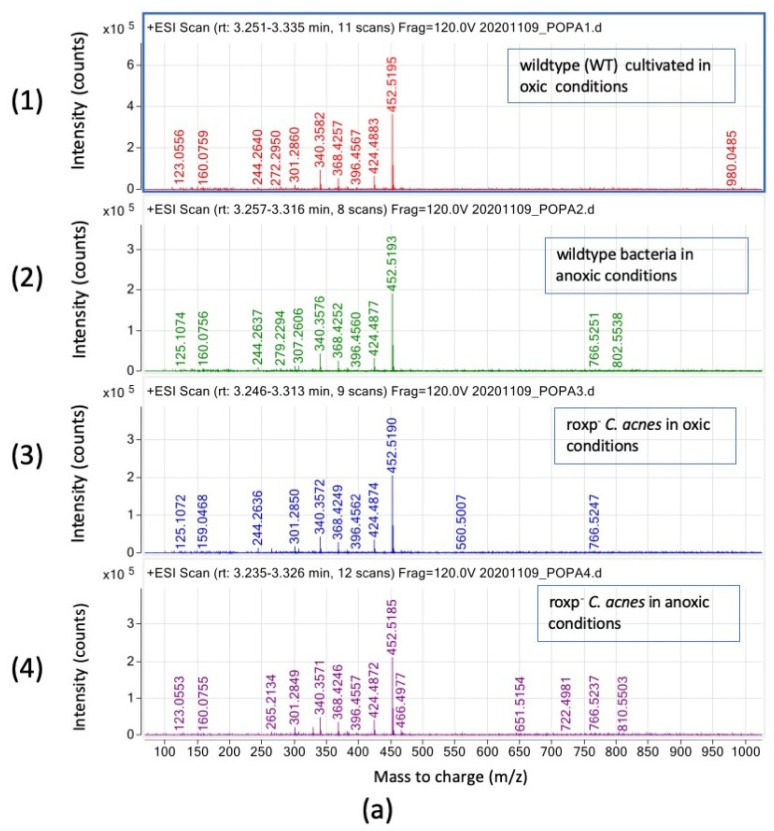

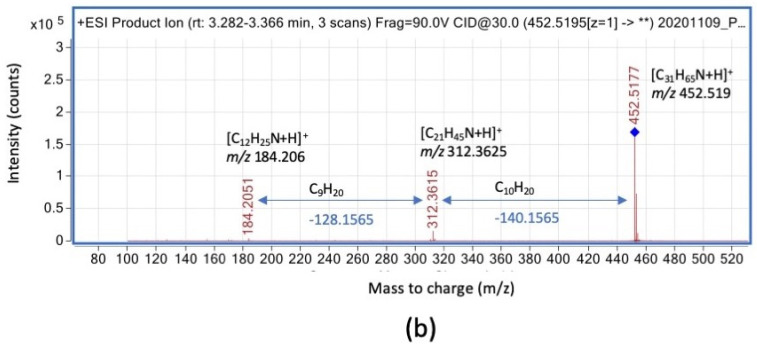
Ceramide species detection in *C. acnes* by SFC-HRMS in positive mode. Intensity (count) versus mass-to charge (*m*/*z*). (**a**) Samples analyzed are wildtype bacteria cultured in oxic (1) or anoxic (2) conditions, compared to the *roxp*^−^ isogenic mutant cultured in oxic (3) or anoxic (4) conditions after SFC-HRMS between 3.2 and 3.4 min. (**b**) MS2 spectra of the precursor ion at *m*/*z* 452.5177 (see the blue diamond) in positive mode with a collision energy of 30 eV (detail in blue color for the size of the fragments).

Finally, we evaluated the effect on other carbonated lipid species eluting between 3.2 and 3.4 min, representing ceramides (Figure 7a,b). For the species for *m*/*z* between 123.05 and 452.52, minor differences could be visualized. The analysis of the most abundant *m*/*z* ion (452.51) of ceramides species of bacteria samples (Figure 7b) corresponds to a ceramide of C28:2, leading to two characteristic peaks at *m*/*z* 312.36 (esterified fatty acid) and *m*/*z* 184.20 (sphingoid base) after fragmentation.

## 4. Discussion

Carbonylation is an important hallmark of oxidation of proteins. We therefore investigated the carbonylation content in *C. acnes* exposed to different oxic conditions, to study the effect of the antioxidant RoxP. Surprisingly, though a trend, we could not see a significant difference in carbonylation attributed to the presence of RoxP. Due to the fact that RoxP is the most abundant protein in the secretome, and due to the high carbonylation level of RoxP itself (“RoxP”, Figure 1), the higher carbonylation level seen in the wildtype secretome as compared to the *roxp* isogenic mutant is likely based on carbonylation of RoxP, indicating that RoxP is one of the dominant carbonylated proteins in the secretome compartment. This agrees with the suggestion that it is a sacrificial molecule for extracellular redox stress, as it is prone to oxidative stress itself, while more vital macromolecules are protected. This latter hypothesis will, however, need further studies to be confirmed. Such hypotheses would also indicate that RoxP is not a classical antioxidant molecule or enzyme, but mediates its effect through the presence of highly accessible amino acids prone to oxidation. This mechanism would fit with RoxP acting in synergy with the other classical antioxidant systems in bacteria, and, as such, it would partly be redundant. This could explain why we only see a trend in reduced protein carbonylation in the presence of RoxP during the experimental settings applied here.

Bacteria contain a wide range of both enzymatic and non-enzymatic defense systems (primarily thiols, but also ascorbate, tocopherols and carotenoids) against oxidants. The systems include enzymes that remove superoxide radicals (superoxide dismutases; to give molecular oxygen and hydrogen peroxide) and enzymes that remove hydrogen peroxide and, to a lesser extent, other peroxidases (multiple catalases, gluthathione peroxidases, peroxiredoxins and thioredoxin peroxidases) [18]. Bacteria are also well endowed with species that regenerate the reducing equivalents (NADPH and thiols) required by these enzymes (e.g., glutathione reductases and glutaredoxins to recycle the oxidized glutathione generated by glutathione peroxidases). Bacteria also contain multiple enzymes (e.g., thioredoxin/thioredoxin reductases) that reduce oxidized proteins and low-molecular mass thiols [19]. A number of other peroxidase enzymes (e.g., cytochrome C peroxidase and chloride peroxidase) also consume hydrogen peroxide with concomitant oxidation of low-molecular mass or metalloprotein (cytochrome) cofactors. Specialized flavohemoglobins also control nitric oxide levels [20]. The majority of these systems are located intracellularly and, as with eukaryotes, extracellular antioxidant defenses are more limited, and these are less efficient. In such circumstances, secreted RoxP may play a key role in removing excess oxidants via direct oxidant scavenging. This is likely to be of particular importance in circumstances when there is an absence or depletion of the reducing equivalents needed to maintain other oxidant-removing enzymatic systems.

Concerning the free fatty acid profiles, it was observed that the ions of *m*/*z* 255.23 are more prevalent in RoxP-containing strains, regardless of culture conditions (e.g., oxic vs. anoxic) and whether chains of FA with C12:0 to C26:0 were found. In respect of fatty acids content in GD and TG, we found a very long FA of C37:0 in DG as reported in the literature [16]. The existence of DG with ester link and O-20:0/O-20:0 was reported and we found the presence of very long FAs (C36:0, C37:0 and C37:1) (Table S2) in the composition of DG, which were both likely to be stabilizing the bacterial structure and acting as energy sources [16].

In the *roxp*^−^ mutant, the DGDG species increased in the relative intensity of the ions (*m*/*z* 896.59 to 930.58) compared to the wt bacteria, meanwhile the relative intensity of the DGDG species from *m*/*z* 937.54 to 953.66 were reduced in the *roxp*^−^ mutant, while the relative intensity of the *m*/*z* ion 910.51 remained constant in both the *roxp*^−^ mutant and wt bacteria, stressing the impact of not only the environmental input on shaping the lipidome, but also that of bacterial proteins (e.g., antioxidants).

The MS spectra of the DG and TG composition show a dependence of the presence of the oxygen atmosphere on the de novo synthesis of the glycerides (MG, DG and TG) using glycerol. The metabolic activity of the diacylglycerol acyl transferase DGAT1 is dependent on the oxygen input and the % of the MG (monoglyceride). It shows a slow accumulation of MG in wildtype (WT) in oxic atmosphere and very low MG amount in the absence of oxygen in anoxic conditions, and even less MG synthetized in anoxic conditions in *roxp*^−^ *C. acnes*.

Concerning the DGs presence in the samples, it was shown that the DGs contained very long fatty acids (ranging from C29:0 to C37:0) but the ion at *m*/*z* 458.34 was detected only in wildtype bacteria grown under oxic conditions. Meanwhile, the triglycerides contained only very long fatty acids ranging from C19:0–C33:0. It was observed in TGs, that the relative intensities of the ions at *m*/*z* 391.28 and *m*/*z* 647.46 are increased in bacteria cultured under anoxic conditions. Meanwhile, the bacteria lacking the antioxidant RoxP have a significantly lower relative intensity TG.

Previously, it was shown that *C. acnes* produces a biofilm containing short fatty acids, which interact with the skin microbiome ensuring its homeostasis [21]. From our results on fatty acids analysis of *C. acnes* wildtype cultured in oxic versus anoxic conditions, and the *roxp*^−^ isogenic mutant cultured in oxic versus anoxic conditions, we observed that the fatty acid prevalence in free form is C12:0 to C26:0 back-bone chains. In complex lipids such as DGs and TGs there are ester links with chains from C20:0 to C37:0, and from C19:0 to C33:0, respectively. In DGDGs and SQGDs, they are in acyl form ranging from C29:0 to C37:0. In SQGDs, they are in acyl form with a backbone and were specifically identified as C30:0, C32:0, C33:6, C34:0, C35:0 and C36:6. Accumulation of some ceramide species with very long fatty acids are observed in anoxic conditions mostly in *roxp*^−^ *C. acnes* compared to the wildtype, which could be a link to the stress cultivation conditions, knowing that ceramide accumulation is a reference for cellular stress and apoptosis [22]. These data show the plasticity of lipids synthesis machinery and the vital dependence of the environmental culture conditions.

## 5. Conclusions

We observed that the carbonylated protein content in *C. acnes* is not significantly affected by environmental oxic atmosphere, nor by prevalence of antioxidant systems in the bacteria. It should be mentioned that *C. acnes* do not solely rely on RoxP for protection against reactive oxygen species, but have several other systems, including catalase and SOD. Furthermore, the fatty acids backbone in FFA contained mainly C12:0–C26:0 in hydroxy and acylated shape, in DG from C29:0–C37:0, in TG from C19:0–C33:0, and in DGDG/SQDGs from C29:0–C37:0 demonstrating the dependence of de novo synthesis of lipids by RoxP.

In conclusion, we have shown that not only do oxidative environments play an important role in affecting the oxidative status of bacterial macromolecules, but also that the presence of the bacterial antioxidant RoxP is key in shaping the lipidome and thus one significant part of the interaction surfaces between the host–microbe. It is thus imperative to investigate these bacterial antioxidant systems further, to better understand their role in host–microbe interactions and the development of pathological conditions.

## Figures and Tables

**Table 1:** Percentage (%) distribution of the compounds in the free fatty acid fractions from the HPTLC plate (Figure 2b).

Sample	Acyl FFA (%)	FFA (%)	OH-FFA (%)
1-wt bacteria (oxic)	21.70	76.40	2.00
2-wt bacteria (anoxic)	45.10	54.70	0.20
3*-roxp*^−^ bacteria (oxic)	52.70	36.20	1.10
4-*roxp*^−^ bacteria (anoxic)	37.70	61.50	1.80

**Table 2:** Area under the curve for the *m*/*z* of DGDG peaks for the four samples eluted at 12.85 min. Numbers indicate area under the curve for individual MS spectra (spectra not shown).

DGDG (*m*/*z*)	910.6112	915.5674	924.6276	929.5833
wt bacteria (oxic)	252,476	117,254	62,233	29,774
wt bacteria (anoxic)	191,071	88,784	67,414	31,552
*roxp*^−^ bacteria (oxic)	108,911	51,809	116,441	53,098
*roxp*^−^ bacteria (anoxic)	90,252	48,843	95,145	50,482

## Data Availability

All data are supplied in the article, as Figures, Tables or Supplementary Data.

## Supplementary Materials

The following supporting information can be downloaded at: https://www.mdpi.com/article/10.3390/microorganisms11092260/s1, Figure S1: Lipids detection in Cutibacterium acnes by SFC-HRMS in positive mode as a function of the retention time (min); Figure S2: HPTLC of the mono-, di- and triglycerides, Table S1: Identified free FA types, Table S2: Identified FA types in diglycerides, Table S3: Identified FA types in triglycerides, Table S4: SQDG *m*/*z* after MS analysis of the 13.3–13.6 min area, and Table S5: SQDG *m*/*z* 805.5 and DGDSG *m*/*z* 910.6 analysis of the 13.3–13.6 min area.
