# Supplementary material for: Oxygen Exposure and Tolerance Shapes the Cell Wall-Associated Lipids of the Skin Commensal Cutibacterium acnes
Source: Microorganisms. 2023 Sep 8;11(9):2260. doi: 10.3390/microorganisms11092260 (PMC10534455; doi:10.3390/microorganisms11092260)
Supplement: Supplementary file 1 [file microorganisms-11-02260-s001.zip › microorganisms-2542725-supplementary.pdf]

Supplementary results

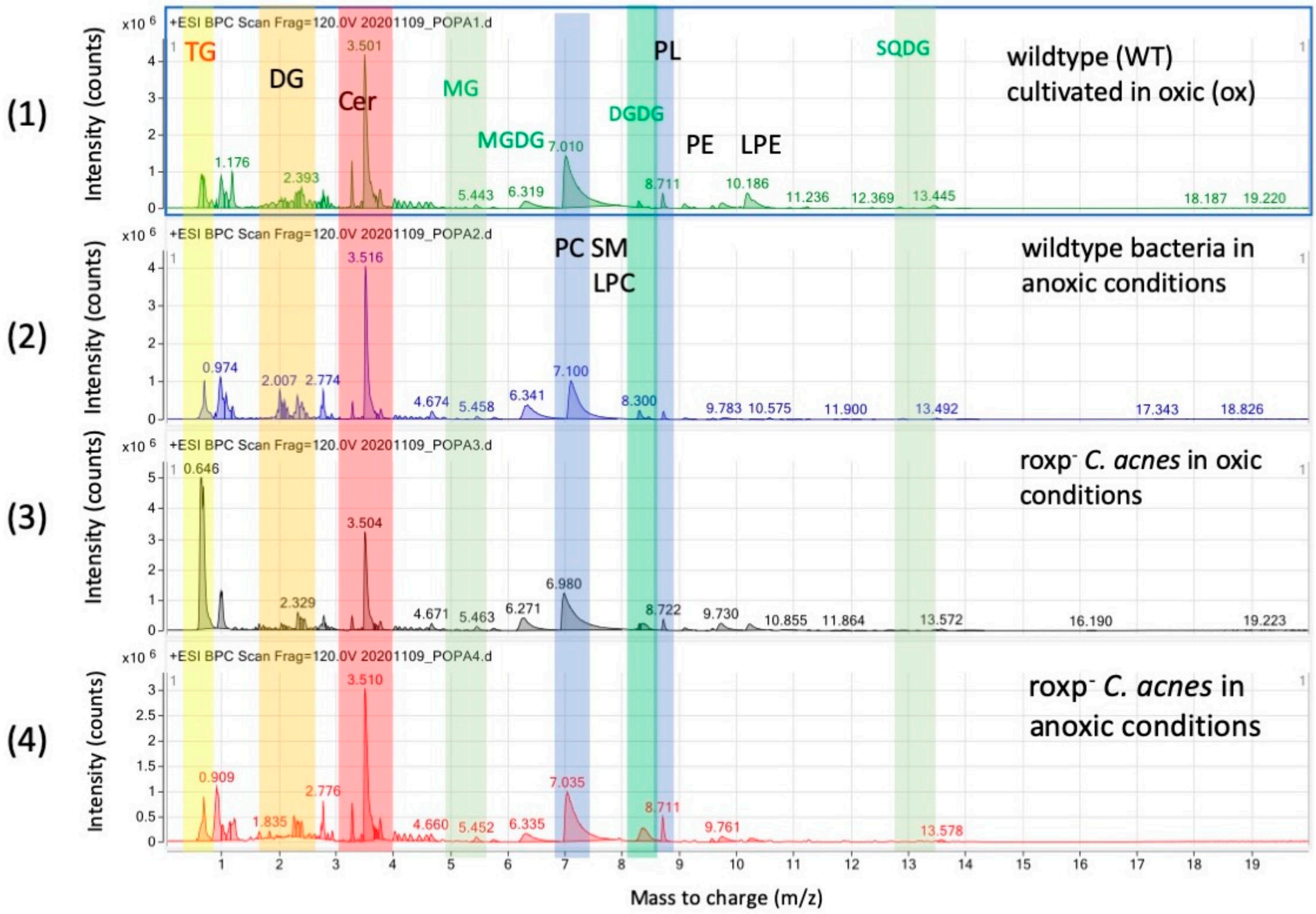

Figure S1. Lipids detection in *Cutibacterium* acnes by LC-MS in positive mode in function of the retention time (min). The lipidome of 1) wildtype bacteria in oxic conditions, 2) wildtype bacteria in anoxic conditions, 3*)* *roxp- C. acnes* in oxic conditions, and 4) *roxp- C. acnes* in anoxic conditions.

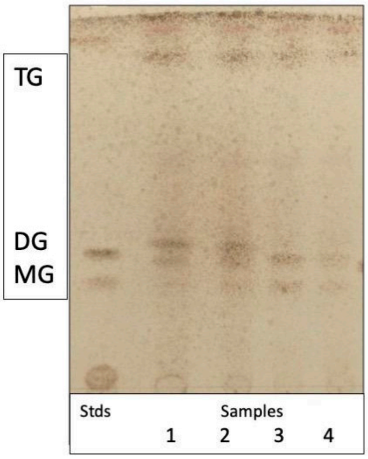

Figure S2. HPTLC of the mono-, di- and triglycerides from all samples (1) wildtype bacteria cultured in oxic (2) wildtype bacteria cultured in anoxic conditions, (3) *roxp-* isogenic mutant cultured in oxic (4) *roxp-* isogenic mutant cultured in anoxic conditions. Spotted amount of the samples is 50% of the whole amount. Spotted standard of Fatty acid is 5 µl (2 mg/ml), revelation in Cu acetate [12].

Table S1. The Free Fatty Acids (FA) Type present in the 4 samples

(Theo- *m*/*z* theoretical, *m*/*z* identified in negative MS)

FA	(*m*/*z*) Theo	(*m*/*z*)
C12:0	199.1698	199.1694
C14:0	227.2007	227.2010
C15:0	241.2164	241.2160
C16:0	255.2320	255.2321
C16:1	253.2163	253.2167
C17:0	269.2477	269.2480
C17:1	267.2320	267.2321
C18:0	283.2633	283.2632
C18:1	281.2477	281.2479
C18:2	279.2320	279.2320
C19:0	297.2790	297.2788
C20:0	311.2946	311.2944
C20:1	309.2789	309.2779
C21:0	325.3103	325.3087
C22:0	339.3259	339.3255
C23:0	353.3416	353.3414
C24:0	367.3572	367.3572
C25:0	381.3729	381.3723
C26:0	395.3885	395.3880

Table S2. The Fatty Acids (FA) Type present in the diglycerides in the 4 samples

(Theo- *m*/*z* theoretical, *m*/*z* identified in positive mode MS)

FA	(*m*/*z*) Theo	(*m*/*z*)
C29:0	544.4933	544.4940
C30:0	558.5103	558.5090
C31:0	572.5258	572.5250
C32:0	586.5413	586.5413
C33:0	600.5571	600.5560
C34:0	614.3573	614.5720
C36:0	642.6035	642.6028
C37:1	654.6036	654.6028
C37:0	656.6183	656.6185

Table S3. The Fatty Acids (FA) Type present in the triglycerides in the 4 samples

(Theo- *m/z* theoretical, *m/z* identified in positive mode MS)

FA	(*m/z*) Theo	(*m/z*)
C19:0	418.3169	418.3174
C22:0	460.3638	460.3642
C24:0	488.3951	488.3958
C25:0	502.4108	502.4106
C26:0	516.4264	516.4273
C27:0	530.4421	530.4432
C28:0	544.4577	544.4589
C29:1	558.4734	558.4747
C30:0	572.4890	572.4896
C31:0	586.5047	586.5054
C33:0	614.5360	614.5364

Table S4. SQDG in the 4 samples *m/z* found by SFC-HRMS analysis of 13.3 – 13.6 min area

(Theo- *m/z* theoretical, *m/z* identified in positive mode MS)

SQDG	(*m/z*) Theo	(*m/z*)
C30:0	789.48	789.48
C31:0	791.40	791.47
C32:6	805.42	805.48
C33:6	819.43	833.51
C34:6	833.45	833.51
C34:0	826.57	826.58
C35:6	842.51	842.57
C36:6	856.53	856.59

Table S5. SQDG *m/z* 805.5 and DGDSG 910.6 after positive mode MS analysis of 13.3 – 13.6 min area (ref [15]**)**

Sulfoglycolipid, *m/z*	Lipid Species (C:N) Carbons atoms: N double bounds	FA acyl chain
805.5	SQDG(33:1)	17:1/16:0
910.6	DGDG (32:0)	16:0/16:0
